# Pivotal Role of Inosine Triphosphate Pyrophosphatase in Maintaining Genome Stability and the Prevention of Apoptosis in Human Cells

**DOI:** 10.1371/journal.pone.0032313

**Published:** 2012-02-27

**Authors:** Miriam Rose Menezes, Irina S.-R. Waisertreiger, Hernando Lopez-Bertoni, Xu Luo, Youri I. Pavlov

**Affiliations:** Eppley Institute for Research in Cancer and Allied Diseases, Nebraska Medical Center, Omaha, Nebraska, United States of America; Karolinska Institutet, Sweden

## Abstract

Pure nucleotide precursor pools are a prerequisite for high-fidelity DNA replication and the suppression of mutagenesis and carcinogenesis. ITPases are nucleoside triphosphate pyrophosphatases that clean the precursor pools of the non-canonical triphosphates of inosine and xanthine. The precise role of the human ITPase, encoded by the *ITPA* gene, is not clearly defined. *ITPA* is clinically important because a widespread polymorphism, 94C>A, leads to null ITPase activity in erythrocytes and is associated with an adverse reaction to thiopurine drugs. We studied the cellular function of ITPA in HeLa cells using the purine analog 6-N hydroxylaminopurine (HAP), whose triphosphate is also a substrate for ITPA. In this study, we demonstrate that *ITPA* knockdown sensitizes HeLa cells to HAP-induced DNA breaks and apoptosis. The HAP-induced DNA damage and cytotoxicity observed in *ITPA* knockdown cells are rescued by an overexpression of the yeast ITPase encoded by the *HAM1* gene. We further show that *ITPA* knockdown results in elevated mutagenesis in response to HAP treatment. Our studies reveal the significance of ITPA in preventing base analog-induced apoptosis, DNA damage and mutagenesis in human cells. This implies that individuals with defective ITPase are predisposed to genome damage by impurities in nucleotide pools, which is drastically augmented by therapy with purine analogs. They are also at an elevated risk for degenerative diseases and cancer.

## Introduction

The human genome is constantly attacked by exogenous or endogenous DNA damaging agents. An accumulation of DNA damage increases genome instability and mutagenesis, which predisposes cells to neoplasia, as well as degenerative diseases [1], [2]. A prominent cause of endogenous DNA damage decreasing the fidelity of DNA replication is contamination of the nucleotide precursor pool with non-canonical nucleotides [3], [4]. These contaminants of the precursor pool include deoxy- and ribonucleoside triphosphates of inosine (ITP/dITP), xanthine (XTP/dXTP), 8-oxo-guanine (8-O-GTP/8-O-dGTP) and others, generated either as byproducts of cellular metabolism or by deamination or oxidation of bases in natural nucleotides. Non-canonical nucleotides contain analogs of the normal nitrogen bases (base analogs), which gives some of them the unique property of ambiguous base pairing during replication [5], [6], [7]. Incorporated base analogs in DNA are repaired by the cellular repair systems, which can result in the accumulation of DNA breaks [8], [9]. If base analogs in DNA escape the repair systems, their capacity for ambiguous base pairing will lead to the accumulation of mutations in the subsequent replication rounds [10], [11]. Taking into consideration the harmful effects of base analog incorporation, it is not surprising that cells have developed elaborate enzymatic systems that protect from base analog-induced DNA damage [12], [13]. These systems function at two levels. The first level involves the interception of non-canonical nucleotides in the precursor pool and their cleavage into di- or monophosphates. The second level involves detection of improper bases after incorporation and their direct removal from DNA. The former is achieved by a class of enzymes called nucleoside triphosphatases (NTPases) [3]. One such NTPase is evolutionary conserved Inosine Triphosphate Pyrophosphatase (ITPA) [14].

ITPA is a human ITPase, whose function is to cleave inosine triphosphate (ITP) and xanthine triphosphate (XTP) as well as their deoxyribose forms into monophospates. This prevents the incorporation of the nucleotide inosine (dITP), which contains the base analog hypoxanthine, and dXTP into DNA [15]. *ITPA* is expressed in many human tissues [15], [16]. The importance of ITPases is underscored by severe genome instability phenotypes caused by deletion of the *ITPA* homologs in bacteria, yeast and mice. A mutant of the bacterial ITPase, *rdgB*, is synthetically lethal in combination with defects in recombination [8], [9], [17]. The deletion of the budding yeast ITPase, *HAM1*, results in a drastic elevation of mutagenesis induced by the model purine base analog hydroxylaminopurine (HAP) [18]. The most severe phenotype for ITPase deletion is observed in mice. The majority of the progeny with ITPase knockout (genotype *Itpa^−/−^*) are inviable [19]. The mice that survive suffer from growth retardation and die before weaning from cardiac failure. Fibroblasts obtained from the ITPase knockout mice accumulated DNA single-strand breaks and chromosomal abnormalities [20]. Therefore, in bacteria, yeast and mice, the ITPase function plays an important role in maintaining genomic integrity.

The precise cellular function of the human ITPase, *ITPA*, is not clearly defined. There is a polymorphism in the *ITPA* gene in the human population. Several alleles cause atypical ITPase activity [21], [22], [23]. Clinically, the most relevant polymorphism is the *ITPAc.*94C>A missense mutation that results in a substitution of proline with threonine at position 32 (P32T). The allelic frequency of this mutation ranges from 5 to19%, with the highest frequency found in the Asian population [22]. Homozygotes for the P32T mutation have no ITPase activity in erythrocytes, whereas heterozygotes have approximately one-fourth ITPase activity. Although ITPA deficiency in humans appears to be benign, the administration of thiopurine therapy leads to adverse drug reactions in these individuals [21], [24], [25].

To analyze the role of ITPA in human cells, we forced nucleotide pool contamination by 6-hydroxyadenine (hydroxylamonipurine, abbreviated HAP). The dHAPTP is as good a substrate for ITPA as ITP or XTP [26]. HAP is a potent mutagen that can mispair with C or T and induce GC to AT and AT to GC transitions [27], [28]. Unlike most mutagens, HAP in non-recombinogenic in yeast and its mutagenic action is independent of translesion synthesis DNA polymerase, Pol ζ [29], [30]. Nevertheless, it is clastogenic in mammalian cells [31]. Most likely, HAP is activated to deoxynucleoside triphosphate by a combination of salvage and de novo purine biosynthesis pathways [26], but definite genetic identification of the responsible enzymes has only been obtained for the first step, conversion of the base to HAPMP by phosphoribosyltransferases in bacteria and yeast (Stepchenkova and Schaaper, personal communication and [32]). The dHAPTP is readily incorporated into DNA by the replicative DNA polymerases of bacteria and eukaryotes [27], [33]and is repaired in bacteria by the same systems as dITP and dXTP [9], thereby enabling us to extrapolate the results obtained with HAP to natural base analogs. Thus, the use of HAP provides us with a good tool to investigate the protective effects of *ITPA* against nucleotide pool contamination.

In this study, using the cervical carcinoma cell line HeLa and HAP as a model, we demonstrate that *ITPA* knockdown sensitizes human cells to base analog-induced DNA breakage, mutagenesis and apoptosis. These phenotypes can be rescued by overexpressing the yeast ITPase, *HAM1*, in the *ITPA* knockdown cells. Our data suggest that *ITPA* plays a critical role in protecting human cells against the cytotoxic, genotoxic and mutagenic effects of base analogs. This implies that individuals with defective ITPase are at an elevated risk for degenerative diseases and cancer.

## Results

### HAP incorporation into DNA of HeLa cells

**Figure 1:**
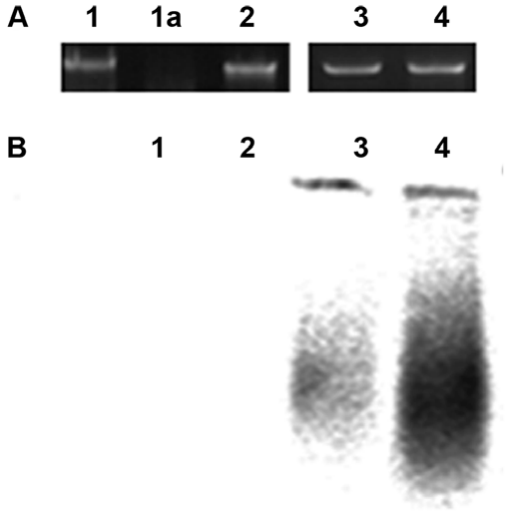
HAP treatment leads to the appearance of EndoV sensitive sites in HeLa DNA. We extracted genomic DNA from HeLa cells grown with or without HAP. Treatment of this DNA with bacterial EndoV creates 3′ nicks, which are substrates for nick-translation (BioProbe® Nick translation kit with bio-16-dUTP (Enzo Life Sciences)) as described in Materials and Methods. A. Agarose gel electrophoresis of nick-translated DNA from HeLa cells. 1- from untreated cells; 1a – from untreated cells digested with DNase; 2 – from cells grown in 2.64 mM HAP; 3- from untreated cells, DNA incubated with Endo V; and 4 - from cells grown in 2.64 mM HAP, DNA incubated with Endo V. B. Detection of newly synthesized biotinylated DNA separated by alkaline agarose electrophoresis. 1- from untreated cells; 2 – from cells grown in 2.64 mM HAP; 3- from untreated cells, DNA incubated with Endo V; and 4 - from cells grown in 2.64 mM HAP, DNA incubated with Endo V.

It is known that hypoxanthine bases accumulate at a detectable level in RNA and in DNA in *Itpa* knockout mice [19], [20]. To find whether HAP is present in DNA of treated HeLa cells, we studied the appearance of endonuclease V-cleavable sites. HAP in DNA is recognized by the product of the bacterial *nfi* gene, EndoV protein [9]. The enzyme cuts the second bond 3′ to the modified base and leaves free 3′ OH groups [34], [35]. Such DNA will be a substrate for nick translation and therefore, the incorporation of label by *E. coli* DNA polymerases I would be proportional to the quantity of such nicks [36]. We found that the number of EndoV cleavable sites tremendously increases in DNA isolated from HeLa cells grown in the presence of HAP (Fig. 1). This means that after 24 hours there is a substantial proportion of HAP in DNA, which was not removed by DNA repair in human cells. We previously detected DNA breaks, presumably being intermediate products of repair of HAP, in the Comet assay after the same 24 hours [37]. While sensitive Comet assay detects some breaks, most HAP is still present in DNA at this time. Massive removal of HAP achieved by EndoV *in vitro* produces a strong signal in nick-translation assay.

### HAP treatment triggers apoptosis in HeLa cells

**Figure 2:**
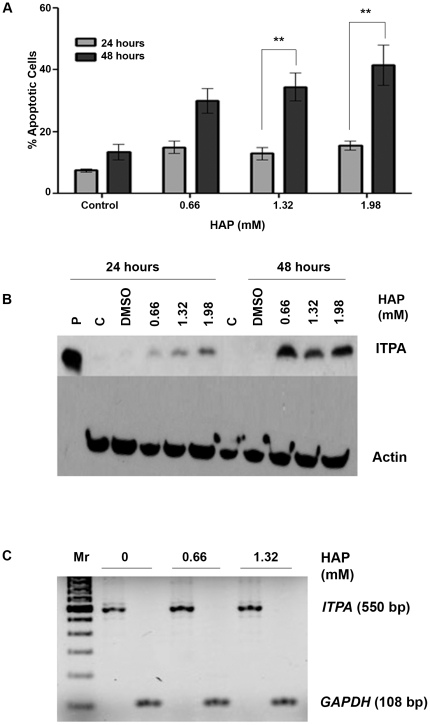
Effects of HAP treatment on apoptosis and ITPA levels in HeLa cells. (A) HAP treatment causes apoptosis in HeLa cells after treatment for 24 or 48 hours. By two-way ANOVA, p_time_ = 0.001 and p_concentration_ = 0.0046. **p<0.01 by Bonferroni multiple comparison post test for column analysis comparing means for 24 hours vs. 48 hours. (B) HAP treatment leads to the increase of ITPA protein levels in HeLa extracts following treatment for both 24 hours and 48 hours. Western blots were performed as described in Materials and Methods. P- pure ITPA protein, C- untreated control, DMSO – solvent only. (C) HAP treatment does not increase levels of ITPA transcripts. The analysis was performed as described in Materials and Methods. HAP treatment was for 24 hours. Mr – 100 bp ladder.

Previously, it has been reported that HAP treatment results in chromosomal fragmentation in human epidermoid cells [38]. This effect appears to be cell line specific, because HAP did not induce a chromosomal catastrophe in HCT116 cells [37]. This suggests that HAP is potentially capable of causing devastating DNA damage in human cells, but this is realized only under certain conditions. We examined whether HAP treatment triggered apoptosis in human cells. To do this, we determined the effects of increasing doses of HAP on the viability of HeLa cells after 24 hours or 48 hours of treatment (Fig. 2A). After staining the cells with Hoechst dye, we enumerated the number of apoptotic nuclei by fluorescence microscopy. No effects of HAP treatment were seen after 24 hours. After 48 hours of HAP treatment, we found that 35% of the cells were apoptotic at a dose of 1.32 mM, while 42% of the cells underwent apoptosis at 1.98 mM. This increase is significant (p<0.01) as compared to the 16% apoptosis observed at these doses after 24 hours of treatment. The two-fold increase in apoptotic cells observed after 48 hours of treatment suggests that HAP is cytotoxic to human cells, but cell divisions are necessary for HAP to exert its cytotoxic effects.

By immunostaining of the whole cells as well as by immunoblot we have shown that HAP treatment causes an induction of the ITPA protein production in HCT116 cells [37]. We confirmed that this was the case for HeLa cells as well. We observed that ITPA protein levels were elevated after 24 hours as well as 48 hours after HAP treatment (Fig. 2B). The mechanism is unclear, but it does not occur at the level of transcription, as demonstrated by RT-PCR (Fig. 2C). The induction of the ITPA protein levels appeared to be more prominent after 48 hours. The increase of ITPA protein production in response to HAP treatment is consistent with the idea of a high demand for ITPA when the precursor pool is contaminated with dHAPTP.

### HAP-induced apoptosis occurs through the intrinsic pathway

**Figure 3:**
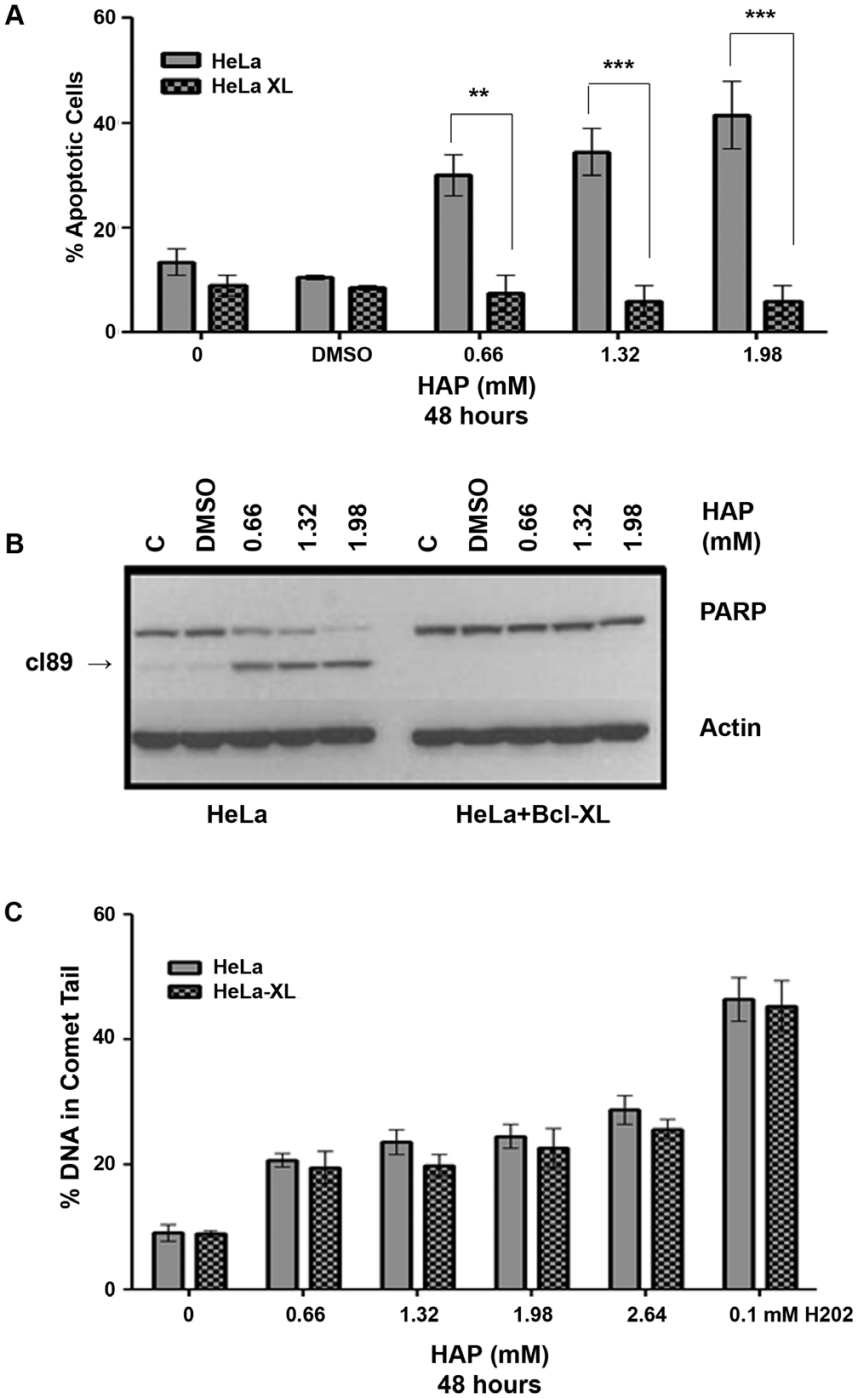
HAP-induced apoptosis occurs through the intrinsic pathway. (A) Protection from HAP-induced apoptosis by overexpression of Bcl-xL. Both HeLa and HeLa-xL cell lines were treated with increasing doses of HAP for 48 hours and the percentage of apoptotic cells was determined by Hoechst staining. By two-way ANOVA, p_cell line_<0.0001 and p_concentration_ = 0.009. **p<0.01,***p<0.001 by Bonferroni multiple comparison post test for column analysis comparing means for HeLa vs. HeLa-xL hours. (B) Confirmation of protection from HAP-induced apoptosis by Bcl-xL overexpression by immunoblot for PARP cleavage. HAP induced dose-dependent cleavage of PARP in regular HeLa cells but not in HeLa cells overexpressing Bcl-xL. PAPR cleavage product is marked as cl_89_. (C) HAP treatment results in similar levels of DNA breaks in HeLa and HeLa+Bcl-xL cell lines (p>0.05).

Apoptosis can occur by two pathways: either the extrinsic pathway that involves death receptors or the intrinsic pathway that occurs through the mitochondria. The intrinsic pathway can be blocked by the overexpression of the anti-apoptotic protein Bcl-xL [39]. To examine which pathway was involved in the case of HAP treatment, we assayed for apoptosis following a 48-hour HAP treatment of HeLa cells overexpressing Bcl-xL (henceforth referred to as HeLa-xL) [40]. As evident from Fig. 3A, the HeLa-xL cells were protected from HAP-induced apoptosis. We confirmed the protection of HeLa-xL cells from HAP-induced apoptosis by performing immunoblots for PARP cleavage, a hallmark of apoptosis [41]. No PARP cleavage (Fig. 3B) was observed in HeLa-xL cells, in a sharp contrast with HeLa cells.

### HAP treatment causes an accumulation of DNA strand breaks prior to the onset of apoptosis

Deletion of the *E. coli* ITPase, *rdgB*, results in the generation of DNA breaks and chromosome fragmentation due to the excision of hypoxanthine by Endo V [8], [9], [17]. We have previously shown that HAP induces DNA breaks in human cells [37]. Most likely, HAP in DNA is processed in a manner similar to the processing of hypoxanthine by either the human homolog of Endo V or some other yet to be identified enzymes. One possible candidate could be the AAG glycosylase, which can excise hypoxanthine [42]. The observation that HAP is capable of inducing apoptosis raised the possibility that the breaks we observe reflect the onset of apoptotic destruction of the nucleus. The HeLa-xL cells provided us with a good tool for distinguishing between the two scenarios. As Bcl-xL overexpression blocks apoptosis, we rationalized that DNA breaks occurring during the repair of HAP incorporated into DNA would not be affected by Bcl-xL overexpression and therefore could be distinguished from DNA breaks caused by the process of apoptosis itself. In the latter case, Bcl-xl overexpression would block the appearance of DNA breaks as apoptosis itself is suppressed. We studied the effect of increasing doses of HAP on the formation of DNA breaks by alkaline comet assay after 24 hours of HAP treatment in both cell lines. In prior experiments, this was a time point where HAP treatment did not cause more than 16% apoptosis. We observed that both cell lines accumulated similar levels of DNA breaks (Fig. 3C). These data suggest that HAP treatment does generate DNA breaks upstream to apoptosis.

### *ITPA* knockdown cells are hypersensitive to HAP-induced apoptosis

**Figure 4:**
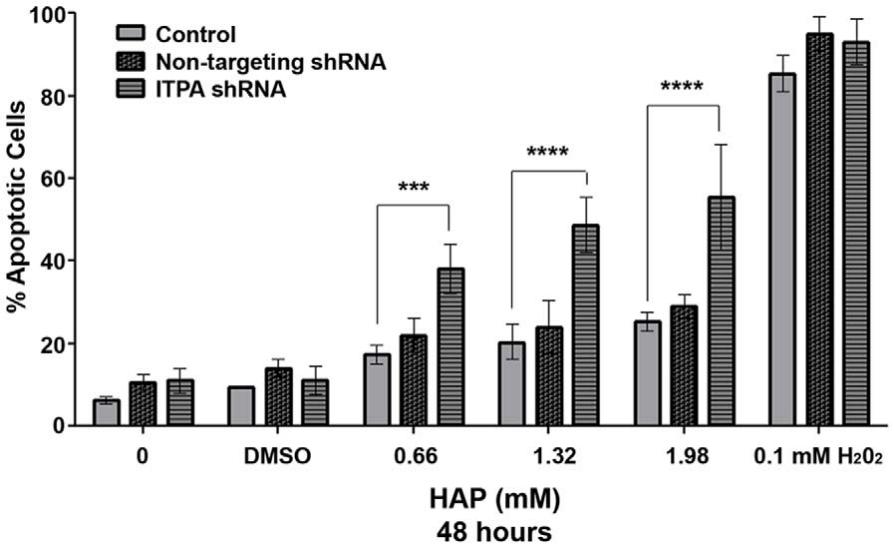
ITPA protects against HAP-induced apoptosis. ITPA knockdown sensitizes cells to HAP-induced apoptosis. As compared to the control and non-targeting shRNA transfected cells, ITPA knockdown cells undergo approximately 30–50% apoptosis upon HAP treatment for 24 hours. Hydrogen peroxide treatment (0.1 mM, four hours) was used as a positive control. The difference between control cells and cells with the *ITPA* knockdown is highly significant (***p<0.001, ****p<0.0001). There was no difference between the control versus the non-targeting cell lines in all HAP doses tested. No significant difference in hydrogen peroxide-induced apoptosis was observed for all three cell lines.

To investigate the role of ITPA in protecting against HAP-induced cytotoxicity, we made stable knockdowns of *ITPA* by transfecting HeLa with plasmids expressing shRNA that targeted the ORF of *ITPA*. We obtained an efficient knockdown of *ITPA* (Fig. S1). The *ITPA* knockdown cells were viable, indicating that it is not an essential gene. Upon treatment with HAP for 24 hours, 30–50% of *ITPA* knockdown cells underwent apoptosis (p<0.001 for 0.66 mM, p<0.0001 for 1.32 mM and 1.98 mM) (Fig. 4). In control HeLa cells, comparable levels of apoptosis were observed only after 48 hours of treatment. No statistically significant differences were observed for the untransfected and non-targeting shRNA transfected controls. Thus, *ITPA* knockdown sensitizes cells to HAP-induced apoptosis. Hydrogen peroxide treatment is known to induce DNA breaks in HeLa cells and subsequently cause apoptosis [43], [44]. Therefore, we used this as a positive control for our apoptosis assays to determine if HAP-induced hypersensitivity to apoptosis was specific for *ITPA* knockdown. We found that hydrogen peroxide treatment caused the same level of apoptosis in all three cell lines. Taken together, our data suggest that *ITPA* plays an important role in protecting HeLa cells against HAP-induced apoptosis.

### Suppression of HAP-induced apoptosis by overexpression of *ITPA* or *HAM1*

**Figure 5:**
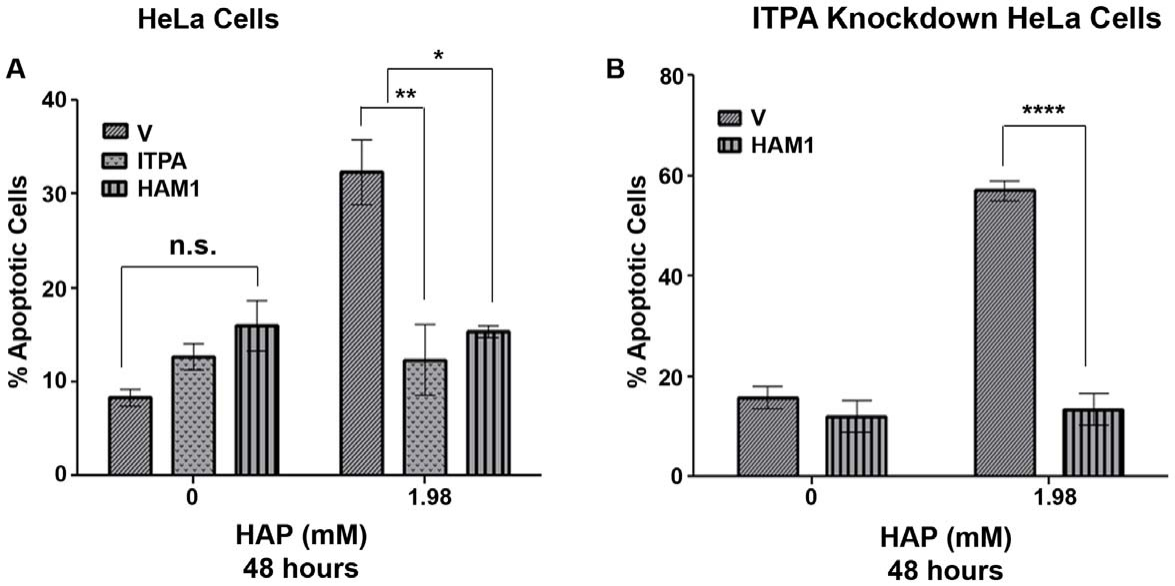
ITPase overexpression suppresses HAP-induced cytotoxicity. (A) Effect of overexpression of the *ITPA* and the gene encoding yeast ITPase, *HAM1*, on HAP-induced apoptosis in HeLa cells. Differences are significant (*p<0.05, **p<0.01). (B) Overexpression of the yeast *HAM1* could rescue *ITPA* knockdown cells from hypersensitivity to HAP-induced apoptosis (****p<0.0001, n.s., not significant).

In prior experiments we found that ITPA protein production was induced in response to HAP treatment (Fig. 2B). This suggested a putative role of *ITPA* in protecting against the harmful effects of HAP. Moreover, we found that *ITPA* knockdown made cells hypersensitive to HAP-induced apoptosis. To further establish the protective role against HAP-induced apoptosis, we determined whether overexpression of *ITPA* or its yeast ortholog, *HAM1*, protected HeLa cells from HAP-induced apoptosis. We transfected HeLa cells with two constructs where ITPase genes were under a strong constitutive promoter. One expresses *ITPA-GFP,* encoding for a fusion of ITPA with the GFP protein, another expresses *HAM1-GFP,* encoding for a fusion of Ham1 with the GFP protein. The production of both fusion proteins was confirmed by immunoblot (Fig. S2A). We compared the apoptotic response at a HAP dose of 1.98 mM for 48 hours. This dose of HAP caused 42% of the cells to undergo apoptosis (Fig. 3A). As compared to the cells transfected with vector alone, *ITPA* overexpressing cells were protected from HAP-induced apoptosis (Fig. 5A, p<0.01). A similar level of protection was also observed in the case of *HAM1* overexpression (p<0.05).

We have shown that the expression of wild-type *ITPA* as well as *HAM1* could efficiently rescue cells from apoptosis caused by HAP treatment, suggesting that the yeast protein functions in the foreign environment (Fig. 5A). Therefore, we rationalized that the high expression of *HAM1* should be able to complement the defects caused by *ITPA* knockdown in those cells as well. We anticipated that *HAM1* would not be inhibited by shRNA targeting *ITPA*. We confirmed the expression of the *HAM1* construct and consequent protein production in the knockdown cells by immunoblot (Fig. S2B). When we treated the transfectants with 1.98 mM HAP for 48 hours, we found that approximately 57% of cells transfected with the vector only underwent apoptosis. However, cells transfected with the vector expressing *HAM1* were resistant, with only 13% undergoing apoptosis (Fig. 4B) (p<0.0001). Thus, *HAM1* complemented the defect seen in the case of *ITPA* knockdown, proving that the differences in the HAP sensitivity of normal and *ITPA* knockdown cells are due to one gene, *ITPA.*

### *ITPA* knockdown leads to elevated levels of HAP-induced DNA breaks

**Figure 6:**
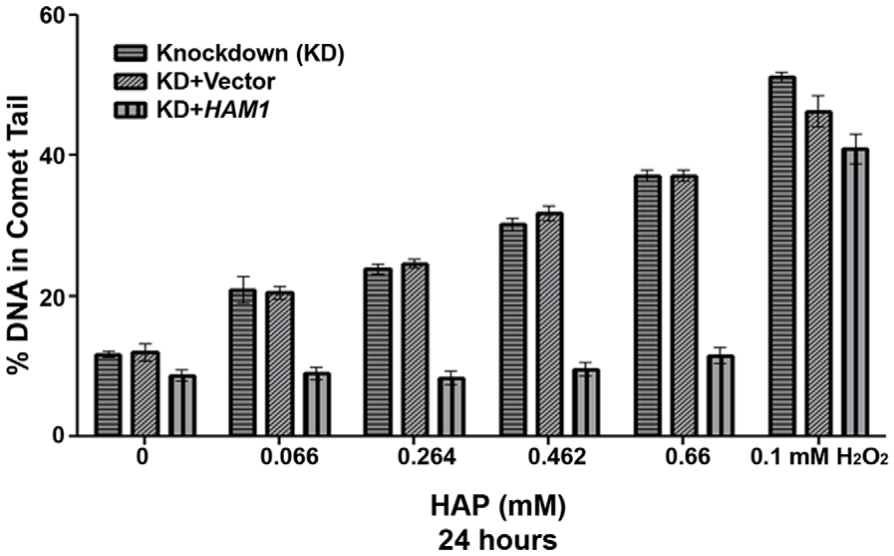
ITPA protects against HAP-induced DNA breaks. Alkaline comet assay data reveals that as compared to the control and non-targeting shRNA-expressing cell lines, ITPA knockdown cells accumulated elevated levels of DNA breaks after 24 hours of treatment with HAP. At high doses of HAP, the sizes of the comet tails in the ITPA knockdown cells were too large to be quantified. The assay was performed at lower doses of HAP treatment in order to obtain measurable comet tails. As compared to the cells with vector, the overexpression of *HAM1* suppressed the accumulation of HAP-induced DNA breaks in the *ITPA* knockdown cells. Statistical differences were measured by one-way ANOVA followed by Dunns multiple comparisons for column analysis. By two-way ANOVA, p = 0.008. Post-test analysis revealed differences (p<0.05) for knockdown versus knockdown+*HAM1* and knockdown+vector versus knockdown+*HAM1*. No difference in levels of DNA breaks was observed for hydrogen peroxide treatment for all three cell lines.

We had previously demonstrated that human dermal fibroblasts with endogenous *ITPA-P32T* (associated with null ITPase activity in erythrocytes, as discussed in the Introduction) accumulated more DNA breaks than the control fibroblast cell line with a wild-type *ITPA* [37]. The availability of *ITPA* knockdown HeLa cells transfected with control vector or vector expressing the yeast *HAM1*gene allowed us to study the effects of HAP in nearly isogenic cell lines. We assayed for levels of DNA breaks by alkaline comet assay at low doses of HAP. At these doses, we did not expect any HAP-induced apoptosis, which would otherwise confound the interpretation of the data since apoptosis itself causes DNA fragmentation. We found that *ITPA* knockdown cells and *ITPA* knockdown cells transfected with the empty vector accumulated more DNA breaks than the knockdown cells overexpressing *HAM1* at low doses of HAP treatment (0.066–0.66 mM) (Fig. 6). At higher doses of HAP, the comet tails in the knockdown cells were too large to be precisely quantified. In the *HAM1* overexpressing a knockdown cell comet tail at higher doses ranged from 13 to 20%, which is slightly less that in the original HeLa, Fig. 3C. No statistically significant difference in levels of DNA breaks was observed in response to hydrogen peroxide treatment in the three cell lines.

### *ITPA* knockdown in HeLa cells elevates HAP-induced mutagenesis

**Figure 7:**
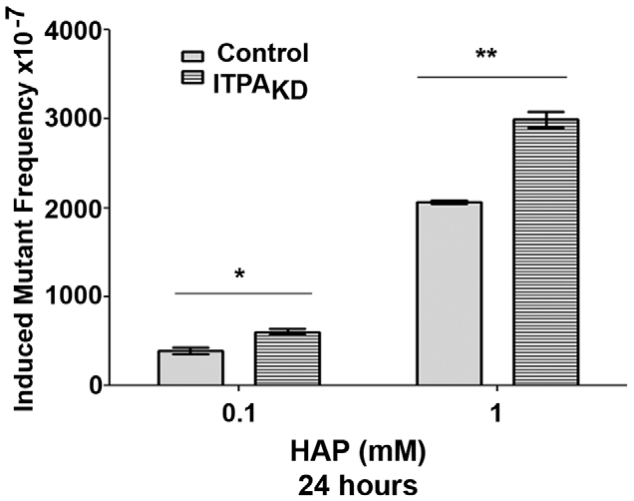
ITPA protects against HAP-induced mutagenesis. The data represent the induced *HPRT* mutant frequency for the HAP-treated control and ITPA knockdown cells. At a low dose (0.1 mM) of HAP treatment for 24 hours the difference between cell lines was not significant but at dose 1 mM ITPA knockdown cells were more sensitive to HAP mutagenesis. (**p<0.01, n.s., not significant).

In addition to triggering apoptosis, we anticipated that contamination of the nucleotide precursor pools with non-canonical nucleotides could lead to elevated mutagenesis. HAP has been shown to be mutagenic and carcinogenic in mammalian cells [45], [46]. It is known that the disruption of the *HAM1* gene in the budding yeast *S. cerevisiae* causes hypermutagenesis in response to HAP treatment [18]. We therefore investigated whether this phenotype would be seen in *ITPA* knockdown human cells as well. We determined that HAP-induced mutant frequencies at the *HPRT* locus (6-thioguanine resistance). The viability of HeLa cell lines with or without ITPA knockdown was not affected by HAP treatment. No statistically significant differences were observed between the untransfected and shRNA-transfected cells in the absence of HAP treatment. HAP was clearly mutagenic for HeLa cells, increasing mutant frequency three-fold at 0.1 mM and 13-fold at 1 mM (Suplementary Table 1). Next we compared HAP-induced mutant frequencies for HeLa and *ITPA* knockdown cells as described in Materials and Methods. We found that *ITPA* knockdown cells were more sensitive to HAP-induced mutagenesis at high doses as compared to the untransfected cells (p<0.01) (Fig. 7). This suggests that ITPA plays a role in the protection from HAP-induced mutations.

## Discussion

An understanding of the role of ITPA in human cells is important because several alleles representing polymorphism in the *ITPA* gene are associated with the onset of thiopurine therapy-related diseases. We probed the function of ITPA by determining whether the enzyme protected HeLa cells against the harmful effects of the model purine analog, HAP.

Previous reports have shown that HAP treatment results in severe genotoxic stress to cells. HAP treatment causes chromosomal fragmentation and mutagenesis in human cells and in Syrian hamster embryo cells [31], [38]. We propose that HAP incorporated into the DNA of human cells (Fig. 1) is being repaired, intermediates of the repair cause breaks and these persisting breaks cause apoptosis.

We found that HAP treatment induced apoptosis in HeLa cells. The manifestation of apoptosis was prominent after 48 hours of treatment (Fig. 2A). This is in contrast to genotoxicants directly damaging DNA, like UV irradiation or hydrogen peroxide, which induce apoptosis within 4–6 hours of treatment [40], [43]. The delayed response suggests that HAP needs to be activated to dHAPTP and incorporated into DNA during replication in order to exert its cytotoxic effects. At present, the exact pathway of HAP activation in human cells is not known. We propose that it could be similar to the hypothetical pathways proposed for bacteria and yeast [26], [32]. As HAP treatment is indicative of the effects of nucleotide pool contamination, our data imply that elevated nucleotide pool contamination and the subsequent incorporation of base analogs into DNA causes apoptosis in human cells.

What is the mechanism underlying HAP cytotoxicity in human cells? Using an analogy to experiments with bacteria, one possibility is the generation of relatively long-lived single strand DNA breaks as intermediates during the repair of HAP in DNA. These breaks are converted to double strand breaks when the replication fork encounters the discontinuity in the template [9], [47], [48].

The incorporation of thiopurines into DNA causes DNA breaks and subsequent apoptosis due to incomplete mismatch repair [49], [50]. An elevated level of the incorporation of 8-oxoG into DNA triggers the accumulation of single-strand DNA breaks, which results in cell death in mouse cells [51], [52]. The situation with 8-OG in mammalian cells is thus different from bacteria, where no chromosomal DNA fragmentation occurs in the *mutT* mutants [53]. It is possible that clastogenicity of base analogs depends on the relative efficiency of the repair systems responsible for their removal from DNA. We propose that HAP-induced apoptosis is caused by persisting DNA breaks. We found that HAP treatment triggered the accumulation of DNA breaks, which later led to apoptosis in HeLa cells but not in HeLa-xL cells. Presumably, these DNA breaks could be caused by the inefficient excision of HAP from DNA by an uncharacterized glycosylase or nuclease. Endo V has been shown to play a key role in this process in *E.coli* lacking the ITPase gene, *rdgB* [9]. Orthologs of the *nfi* gene encoding for endonuclease V have been characterized in mice and found in the human genome [34], [35], [54]. The mouse enzyme was, however, was 50 times less active than the bacterial Endo V. The variants of human enzyme have been purified but no endonuclease activity was detected (Waisertreiger, unpublished, R. Dalhus, personal communication). The exact mechanism of HAP and hypoxanthine repair in humans remains to be determined.

We found that knocking down *ITPA* by shRNA sensitized cells to HAP-induced apoptosis (Fig. 4). The knockdown cells *per se* are viable, thereby indicating that *ITPA* is not an essential gene in human cells. Overexpression of the yeast *HAM1* in the knockdown cells rescued them from the cytotoxic effects of HAP. The knockdown cells accumulated more DNA breaks, which were suppressed in knockdown cells that overexpressed *HAM1* (Figs. 5B and 6). Thus, *ITPA* prevents the accumulation of HAP-induced DNA damage. In passages of mouse embryonic fibroblasts, a spontaneous increase in production of the NUDIX protein, NUDT16, suppressed the genome instability phenotypes associated with *Itpa* deletion, thus suggesting a functional redundancy between *Itpa* and *NUDT16* [20]. Here, we did not observe any redundancy for *ITPA* function. Although NUDIX proteins are found in human cells, it is possible that they are activated under special conditions. It is also possible that *ITPA* knockdown by itself is insufficient to activate the expression of *NUDIX* genes in human cells in a relatively limited number of passages. Another possibility is that the functional redundancy between *NUDIX* genes and *ITPA* is specific to mice. Collectively, we have shown that *ITPA* plays a critical role in preventing HAP-induced apoptosis. This implies that *ITPA* could play a role in protecting against the incorporation of dITP/dXTP as well.

Thiopurines like azathioprine are commonly used immunosuppressive or anti-cancer drugs that exert their cytotoxic effects by being converted into active nucleotides, which are then incorporated into DNA. ITPA is capable of destroying nucleoside triphosphate of 6-MP [55]. A number of reports link chronic immunosuppression/therapy with thiopurines like azathioprine and the onset of therapy-induced cancer. One of the mechanisms by which thiopurines bring about immunosuppression is by causing the death of cytotoxic T lymphocytes, which is elicited by the persistence of DNA breaks [50], [56]. It is plausible that the mechanism of cytotoxicity is similar for thiopurines and HAP. Increased DNA damage can force cells to undergo either apoptosis or senescence in order to prevent the passage of damaged DNA to progeny cells. While apoptosis is critical for tissue homeostasis, increased apoptosis is harmful because it can lead to organ damage and degeneration. Elevated levels of DNA breaks and apoptosis have been implicated as one of the causes of degenerative diseases such as diabetes, arthritis, and cardiac failure as well as neurodegenerative diseases like Alzheimer's disease, Huntington's disease and Parkinson's disease [57], [58]. The increased apoptosis observed in the *ITPA* knockdown cells in response to HAP treatment is clinically relevant because it raises the possibility that individuals with ITPase deficiency could be at risk of developing therapy-induced organ damage and degeneration.

Therapy-induced cancer is a serious side effect of long-term thiopurine-mediated immunosuppression [50], [56]. In transplant recipients, a decade long exposure to thiopurines like azathioprine is associated with the onset of cancers such as leukemia and squamous cell carcinomas. Moreover, there is a putative association between P32T ITPA, prolonged azathioprine therapy and the onset of carcinoma [59]. We have shown that *ITPA* knockdown results in increased HAP-induced mutagenesis. This is in line with our previous findings in yeast wherein HAP treatment in yeast with a *HAM1* mutation resulted in two orders of magnitude higher mutagenesis as compared to their wild-type counterparts [18], [29]. In human cells, however, *ITPA* knockdown did not result in such a dramatic phenotype as was observed with yeast. The situation resembles the discrepancies in magnitude of the effect of the absence of 8-oxoguanine triphosphatase in bacteria (*mutT* is a very strong, up to 1000-fold, mutator [60]) and mice (*Mth^−/−^* cells possess a very weak two-fold mutator phenotype [61]). In spite of this relatively small effect on the mutation rates, the deletion of *Mth1*caused an accumulation of 8-oxoguanine in DNA and resulted in an increase in tumors in mice. In our study, ITPA plays a more prominent role in prevention of apoptosis that mutagenesis caused by HAP, suggesting that different pathways lead to these events. It is also possible that the yeast *ham 1* mutant hypermutability maybe a very special case, because yeast possess neither a molybdenum cofactor-dependent pathway of HAP destruction [62], [63], nor Endo V. Elevated levels of DNA breaks and the increase in HAP-induced mutant frequencies observed in the case of *ITPA* knockdown provide a possible link between *ITPA* deficiency and a predisposition to therapy-related and spontaneous cancer caused by intrinsic base analogs.

**Figure 8:**
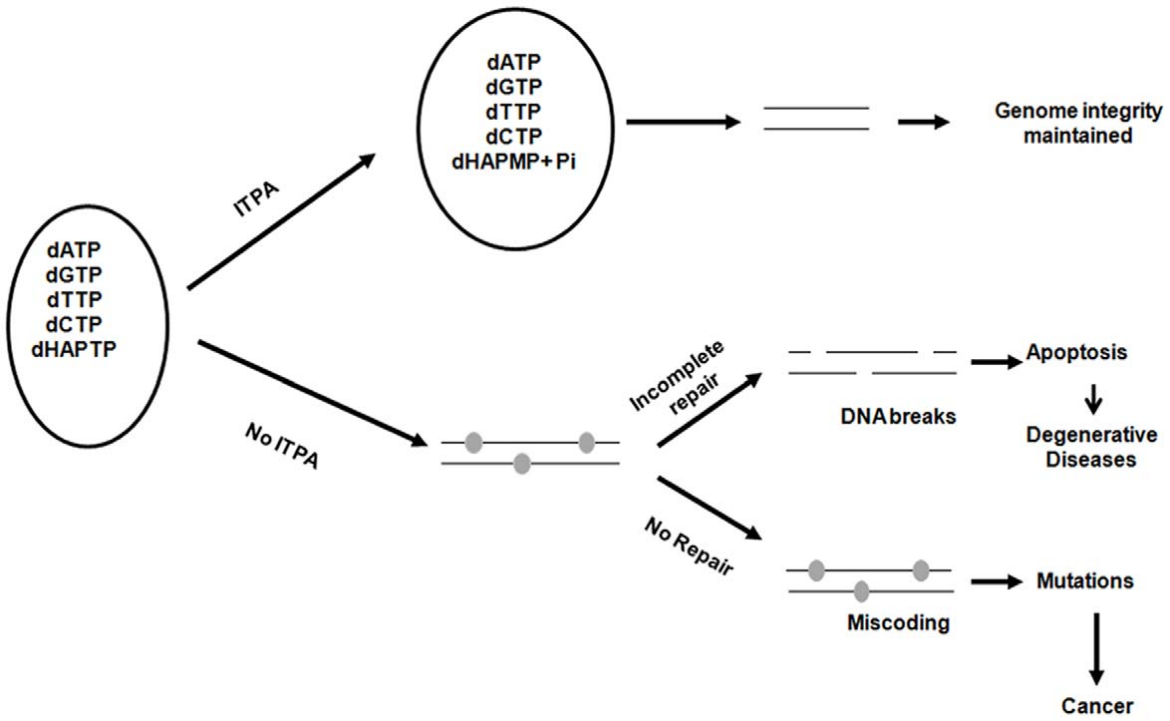
Model for the protective role of ITPA against HAP-induced genotoxicity and mutagenesis. In the presence of functional ITPA, the accumulation of non-canonical nucleotides like dHAPTP is abrogated by the ITPase, thereby preventing their incorporation into DNA. In the absence of functional ITPase, dHAPTP accumulates in the precursor pool and is incorporated into DNA by the replicative DNA polymerases. Grey circles represent HAP accumulation in DNA. Slow excision of base analogs by an unknown nuclease/glycosylase results in the accumulation of single-strand DNA breaks, which triggers apoptosis. Increased levels of apoptosis contribute to the onset of degenerative diseases. In the absence of repair, HAP persists in DNA causing incorrect pairing with T or C, thus leading to the accumulation of mutations, which predisposes individuals to the development of cancer.

We have demonstrated that the elevated nucleotide pool contamination by nucleotides containing HAP, and by extrapolation, endogenous hypoxanthine and xanthine, causes high levels of apoptosis in human cells which, if unchecked, could lead to the onset of degenerative diseases. We have uncovered the critical role of *ITPA* in maintaining the stability of the genome and apoptosis in human cells. Based on the data obtained in this study and concepts generated with model systems, we propose the following model for the role of ITPA in human cells (Fig. 8). In the presence of functional ITPA, the accumulation of non-canonical nucleotides like dITP, dXTP or dHAPTP is prevented due to the ITPase activity. This precludes the accumulation of base analogs hypoxanthine, xanthine or HAP into DNA and helps maintain the genome stability. In the absence of functional ITPase, non-canonical nucleotides accumulate in the precursor pool and base analogs are incorporated into DNA by the replicative DNA polymerases. Repair of base analogs results in the accumulation of DNA single-strand breaks, which are converted to double-strand breaks during replication. This triggers apoptosis and increased levels of apoptosis contribute to the onset of degenerative diseases. In the absence of repair, base analogs persist in DNA, causing errors in replication. This leads to the accumulation of mutations, which predisposes individuals to cancer development. Overall, the findings from this study suggest that *ITPA* is an important factor in the maintenance of genome stability and protection from the onset of therapy-induced degenerative diseases and cancer.

It is also worth mentioning that, in some cases, the defect of ITPA could be a benefit in special situations, because the condition prevents hemolytic anemia in hepatitis C patients treated by ribavirin [64]. This might partially explain why ITPA-P32T allele is retained in population and demands further studies of the role of ITPA and its variants in humans.

## Materials and Methods

### Cells and cell culture

HeLa cervical carcinoma cell line and its derivative overexpressing Bcl-xL (HeLa-xL) were described previously [40]. Both cell lines were cultivated in DMEM (Invitrogen, U.S.A.) containing 10% fetal bovine serum (FBS, Gibco) at 37°C in a 5% CO_2_ atmosphere.

### Chemicals

The base analog 6N-hydroxylaminopurine (HAP) was obtained from MP Biologicals Inc. U.S.A. Stocks of HAP were prepared in DMSO and heated slightly to facilitate dissolution of HAP powder. Hydrogen peroxide (H1009) was obtained from Sigma Co (St. Louis, MO). Doses of HAP used in the experiments ranged from 0.066 mM to 1.98 mM, which corresponded to concentrations of 10 µg/mL to 300 µg/mL.

### Transfection of human cells and generation of stable cell lines

Transfection experiments were carried out using Lipofectamine LTX reagent (Invitrogen, U.S.A.) according to the manufacturer's instructions. Plasmids for the expression of shRNA against *ITPA* (Cat # KH0744P) were purchased from SABiosciences (MD). The shRNA encoded by these plasmids target the ORF of *ITPA*. HeLa cells were transfected by the four plasmids individually and assayed for knockdown efficiency by immunoblot and HAP cytotoxicity by Hoechst staining. As per the instructions provided by the manufacturer, the two plasmids with the highest knockdown efficiency were selected for creating cell lines with stable *ITPA* knockdown. In this case, 1 µg each of plasmids 1 and 2 were used for co-transfection and stable maintenance of plasmids was achieved by selection for puromycin resistance. Stable transfectants were cultivated in DMEM+10% FBS+3 µg/mL puromycin for at least one week before proceeding with further experimentation. *ITPA* stable knockdown typically lasted for about three weeks.

### Plasmid construction

DNA fragments with *ITPA* and *HAM1* were PCR-amplified from previously constructed bacterial expression plasmids [65], [66], [67]. Both ORFs were cloned into the *Eco*RI-*Bam*HI sites of the mammalian expression vector pEGFP-C1 in frame with GFP.

### Antibodies

Antibody against PARP (#9542) was purchased from Cell Signaling. Antibody against β-Actin (A5441) was purchased from Sigma Co (St. Louis, MO). Antibody against GFP (SC9996) was purchased from Santa Cruz Biotechnology Inc. The polyclonal antibody against ITPA was developed in–house [67].

### Immunoblotting

HeLa cells were harvested by scraping with plastic scrapers and pelleted at 1000 g at 4°C. The cell pellets were washed twice to remove traces of medium. Lysates were prepared by resuspending the harvested cell pellets in NP-40 lysis buffer containing 0.1 mM PMSF, 1× HALT protease inhibitor cocktail (Fisher Scientific) and 1 mM sodium orthovanadate. Protein concentrations in the lysates were determined by a Bradford assay (Biorad). Separation of proteins was done by SDS PAGE. The amount of lysate corresponding to 50 µg of protein was boiled in Laemmli's buffer containing 0.1 M DTT and then loaded onto a 4–20% Tris-Glycine gel (Invitrogen, U.S.A.). Resolved proteins were transferred onto a PVDF (Millipore) membrane and blocked in 5% non-fat dry milk/1× PBST for 20 min. Thereafter, membranes were incubated with the appropriate dilution of the primary antibody with shaking overnight at 4°C. Membranes were washed three times in 1× PBST for 10 min each and incubated with appropriately diluted HRP-linked secondary antibody (Fisher Scientific) for one hour at room temperature. The membrane was washed three times in 1× PBST for 15 min each, and signals were detected by Immobilion Western chemiluminescent HRP substrate (ECL, Millipore), according to the manufacturer's instructions.

### RT-PCR

Total RNA was extracted using RNeasy Kit (Qiagen). The cDNA was synthesized from 2 µg of RNA using the Superscript III first strand cDNA synthesis kit (Invitrogen), according to the manufacturer's instructions. The part of the *ITPA* transcript was amplified using the primers 5′-TCATTGGTGGGGAAGAAGATC-3′ and 5′AAGCTGCCAAACTGCCAAA-3′. PCR amplification using these primers gives 550 bp of product. The primers 5′-TCCACCACCCTGTTGCTGTA-3′ and 5′-ACCACAGTCCATGCCATCAC-3′ were used for amplification of the part of the housekeeping gene *GAPDH* transcript to give a product of 108 bp.

### Quantification of the levels of apoptosis

Apoptosis was quantified according to nuclear morphology by using Hoechst 33342 at 1 µg/mL (Molecular Probes). Three different viewing areas were randomly chosen for each experiment. Pictures containing approximately 300 cells were taken for each Hoechst stained viewing area. The percentage of cells undergoing nuclear condensation was calculated for each viewing area. At least three independent experiments were quantified for each data point.

### Alkaline comet assay for assessing accumulation of DNA breaks

Comet assay with HAP was described previously. All of the required chemicals were purchased from Trevigen, Inc. (U.S.A.) [37].

### Determination of the frequency of HAP-induced HPRT mutants

Parent HeLa cells and *ITPA* knockdown cells were plated at a density of 10 million cells per 10 cm dish. Cells were allowed to attach overnight, following which the growth medium was replaced with fresh medium or by medium with the appropriate dose of HAP. Cells were incubated for 24 hours and then the treatment medium was replaced with normal growth medium for 24 hours to allow for phenotypic expression of 6-TG resistant mutants. Cells were then trypsinized and 10^7^ cells were plated onto twenty 10 cm dishes containing growth medium supplemented with 6 µg/mL 6-thioguanine (Sigma, St. Louis, MO) at a density of 5×10^5^ cells per plate. For plating efficiency, cells were plated in three plates with normal growth medium at a density of 5×10^2^ per plate. After two weeks, cells were fixed with 70% ethanol and stained with 5% Giemsa solution in PBS. Colonies were counted macroscopically for plating efficiency. For 6-TG resistance, colonies were counted using an inverted light microscope at 10× magnification. Aggregates of 50 cells or more were scored as a colony. Mutant frequency was calculated using the formula of Glaab and Tyndall (49):
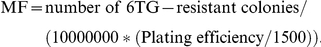
This experiment was repeated three times with three consecutive passages of cells. In every experiment we determined the induced mutant frequency, where the background mutant frequency of untreated cells was subtracted from the frequency of mutants in the HAP treated cultures. Spontaneous mutant frequencies in these experiments ranged from 100 to 500×10^−7^, which is consistent with the published data for HeLa and it derivatives [68].

### Statistical analyses

Each experiment was repeated independently at least twice. All data are expressed as mean +/−SEM of several experiments. Statistical analysis was performed using Graphpad PRISM software (PRISM, CA). Unless otherwise indicated, statistically significant differences between means were estimated using two-way ANOVA to compare concentrations and cell lines. The Bonferroni multiple comparison post-test was used to analyze differences between groups. Values were considered statistically different if the probability of the difference by random fluctuation was less than 0.05.

## Supporting Information

Figure S1 **ITPA knockdown in HeLa cells.** HeLa cells stably expressing shRNA against *ITPA* were immunoblotted for the level of ITPA protein. Cells expressing shRNA against *ITPA* showed an almost complete inhibition of ITPA protein production.
(TIF)

Figure S2 **ITPases overproduction in HeLa cells.** Immunoblot for eGFP in cells transfected with constructs expressing either ITPA or HAM1 as GFP fusion proteins. Cells transfected with vector showed a lower molecular weight band of 27 kDa corresponding to the molecular weight of eGFP. Cells transfected with the *ITPA-GFP* and *HAM1-GFP* showed higher molecular weight bands of approximately 50 kDa, corresponding to the weights of the two GFP fusions with ITPases. We show the immunoblot for eGFP in ITPA knockdown cells. HeLa cells with ITPA knockdown were transfected with constructs encoding for Ham1 as a GFP fusion protein.
(TIF)

Table S1 **Frequencies of spontaneous and HAP-induced HRPT mutants in HeLa cells.**
(DOC)
