# Supplementary figures and images for: Pivotal Role of Inosine Triphosphate Pyrophosphatase in Maintaining Genome Stability and the Prevention of Apoptosis in Human Cells
Source: PLoS One. 2012 Feb 27;7(2):e32313. doi: 10.1371/journal.pone.0032313 (PMC3288088; doi:10.1371/journal.pone.0032313)

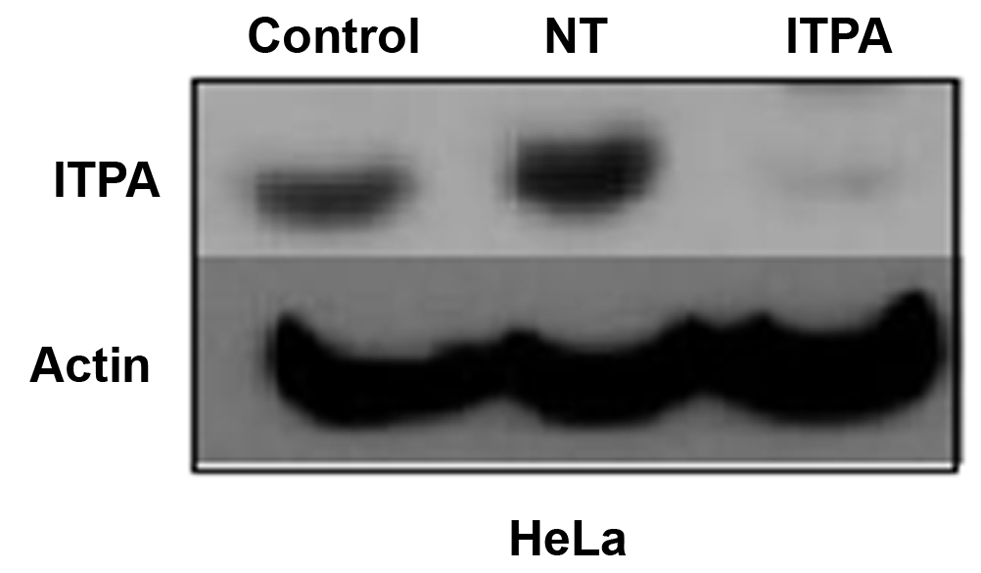

Supplement: Figure S1 — ITPA knockdown in HeLa cells. HeLa cells stably expressing shRNA against ITPA were immunoblotted for the level of ITPA protein. Cells expressing shRNA against ITPA showed an almost complete inhibition of ITPA protein production. (TIF) [file pone.0032313.s001.tif]

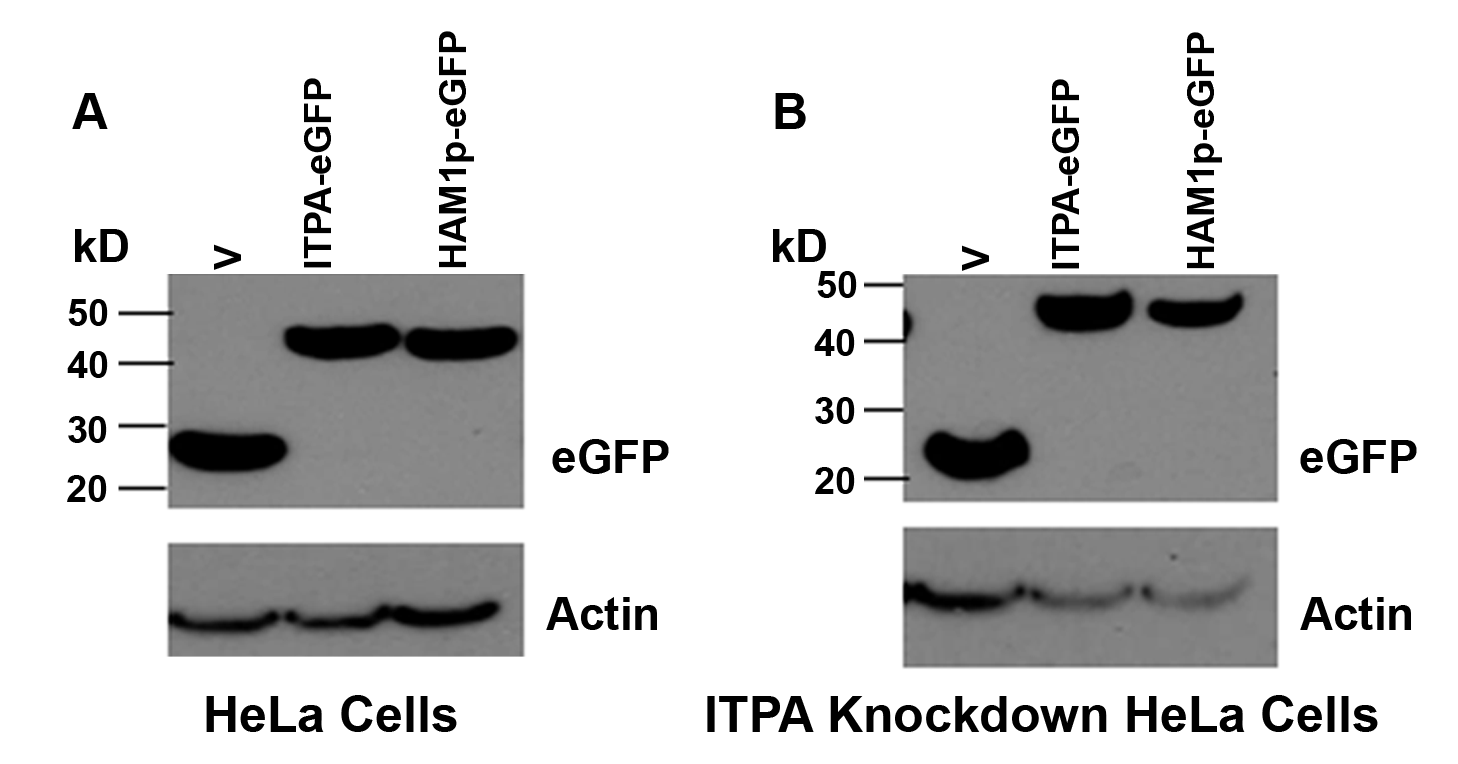

Supplement: Figure S2 — ITPases overproduction in HeLa cells. Immunoblot for eGFP in cells transfected with constructs expressing either ITPA or HAM1 as GFP fusion proteins. Cells transfected with vector showed a lower molecular weight band of 27 kDa corresponding to the molecular weight of eGFP. Cells transfected with the ITPA-GFP and HAM1-GFP showed higher molecular weight bands of approximately 50 kDa, corresponding to the weights of the two GFP fusions with ITPases. We show the immunoblot for eGFP in ITPA knockdown cells. HeLa cells with ITPA knockdown were transfected with constructs encoding for Ham1 as a GFP fusion protein. (TIF) [file pone.0032313.s002.tif]
