# Supplementary material for: Pivotal Role of Inosine Triphosphate Pyrophosphatase in Maintaining Genome Stability and the Prevention of Apoptosis in Human Cells
Source: PLoS One. 2012 Feb 27;7(2):e32313. doi: 10.1371/journal.pone.0032313 (PMC3288088; doi:10.1371/journal.pone.0032313)
Supplement: Table S1 — Frequencies of spontaneous and HAP-induced HRPT mutants in HeLa cells. (DOC) [file pone.0032313.s003.doc]

Supplementary Table 1. Frequencies of spontaneous and HAP-induced HRPT mutants in HeLa cells.

| Treatment | Frequency of 6-TG resistant mutants x 10^-7^ |
| --- | --- |
| none | 164 + 32 |
| 0.1 mM HAP | 560 + 20 |
| 1 mM HAP | 2230 + 50 |
